# When “Normal” Hemoglobin May Not Be Enough: Sex-Specific Anemia Thresholds and Outcomes in Cardiac Surgery

**DOI:** 10.3390/jcm15218632

**Published:** 2026-11-05

**Authors:** Esther Méndez, Elvira Bisbe, Misericordia Basora, Adrián Vizoso, Albert García-Casanovas, Maria J. Colomina

**Affiliations:** 1 Department of Anesthesiology, Bellvitge University Hospital, 08907 L’Hospitalet de Llobregat, Spain; mjcolomina@gmail.com; 2 Neuroscience Research Group—Perioperative Pathophysiology and Pain Unit, Bellvitge Biomedical Research Institute (IDIBELL), 08908 L’Hospitalet de Llobregat, Spain; 3 Perioperative Medicine Research Group, Hospital del Mar Research Institute, 08003 Barcelona, Spain; elvirabisbe@gmail.com (E.B.); avizoso@researchmar.net (A.V.); agarcia8@researchmar.net (A.G.-C.); 4 Department of Anesthesia, Hospital del Mar, 08003 Barcelona, Spain; 5 Department of Anesthesiology, Hospital Clínic de Barcelona, 08036 Barcelona, Spain; coiabasora@gmail.com; 6 Department of Clinical Sciences, Faculty of Medicine and Health Sciences, University of Barcelona, 08907 L’Hospitalet de Llobregat, Spain

**Keywords:** preoperative anemia, hemoglobin, red blood cell transfusion, patient blood management, sex differences, cardiac valve surgery

## Abstract

**Background/Objectives:** Sex-specific anemia criteria classify women with hemoglobin (Hb) 12.0–12.9 g/dL as non-anemic. We assessed whether World Health Organization (WHO) criteria underrecognize red blood cell (RBC) transfusion risk compared with a uniform patient blood management (PBM) threshold of Hb <13.0 g/dL in elective open cardiac valve surgery (CVS). **Methods:** This nationwide retrospective cohort included adults undergoing elective open CVS in 40 Spanish centers (2016–2024), 32 of which reported preoperative Hb. Hb was modeled continuously and in three strata (<12.0, 12.0–12.9, ≥13.0 g/dL). The primary outcome was RBC transfusion; secondary outcomes were postoperative complications, length of stay, and in-hospital mortality within 30 days. Mixed-effects models adjusted for age, comorbidity burden, surgical complexity, valve position, year of surgery, and center. **Results:** Among 29,757 eligible patients, 20,017 had available Hb measurements; 9247 (46.2%) were women, including 2333 (25.2%) with Hb 12.0–12.9 g/dL. In this stratum, 68.0% of women and 52.7% of men received RBC transfusions. The adjusted between-sex probability difference was 15.1 percentage points (95% CI, 11.4–18.9; adjusted odds ratio, 1.85; 95% CI, 1.59–2.16). The categorical sex × Hb interaction was significant (*p* < 0.001), whereas the continuous interaction was not (*p* = 0.486). Sex did not significantly modify the Hb associations with complications or in-hospital mortality within 30 days. **Conclusions:** Women with Hb 12.0–12.9 g/dL had substantial transfusion risk despite being classified as non-anemic by the WHO criteria. Hb <13.0 g/dL may be a pragmatic sex-inclusive trigger for perioperative risk recognition and etiologic evaluation, not a proven therapeutic target.

## 1. Introduction

Women undergoing cardiac surgery receive red blood cell (RBC) transfusions more frequently than men and, in several large cohorts, experience higher short-term morbidity and mortality after adjustment for baseline risk and procedural characteristics [1,2,3]. The mechanisms underlying these differences are multifactorial, but perioperative anemia appears to play an important role. In a nationwide analysis of more than 1.4 million patients undergoing coronary artery bypass grafting, intraoperative anemia mediated a substantial proportion of the excess operative mortality observed in women [3]. These findings raise the possibility that sex-related disparities may be influenced not only by differences in comorbidity and surgical complexity, but also by how preoperative hemoglobin (Hb) reserve is assessed and managed.

Preoperative anemia is common in cardiac surgery and is consistently associated with increased RBC transfusion, acute kidney injury, infection, prolonged hospital stays, and mortality [4,5,6,7]. Its detection and treatment therefore constitute a core component of patient blood management (PBM). Contemporary consensus recommendations advocate systematic anemia screening before major surgery, etiologic evaluation, and treatment when feasible, and they support an Hb concentration below 13 g/dL in both women and men as a pragmatic perioperative definition of anemia [8]. However, although the prognostic association between anemia and adverse outcomes is well established, evidence that short-term pharmacological optimization consistently improves major postoperative outcomes remains less certain [4,9]. Identification of patients at increased risk should therefore be distinguished from evidence that treating all patients to a predefined Hb target necessarily improves clinical outcomes.

The World Health Organization (WHO) defines anemia as an Hb concentration below 12 g/dL in non-pregnant women and below 13 g/dL in men. These thresholds were developed primarily as population-based reference cutoffs rather than as outcome-derived thresholds for patients undergoing surgery associated with expected blood loss. Their applicability to older women and to high-risk surgical populations has consequently been questioned. Women older than 60 years with Hb concentrations of 12.0–12.9 g/dL have shown poorer health-related quality of life than women with higher concentrations [10]. In cardiac surgery, women within this same Hb range—classified as non-anemic according to WHO criteria—have higher transfusion rates and longer hospital stays than women with Hb concentrations of at least 13 g/dL [11]. Subsequent studies have similarly associated this “borderline” or “marginal” Hb range with increased transfusion exposure and, in some cohorts, a higher incidence of postoperative complications in women undergoing major surgery [12,13].

Several physiological factors may increase the clinical relevance of this Hb range in women. For comparable surgical procedures, women generally have a lower circulating blood volume and lower total Hb mass than men, while being exposed to similar absolute surgical blood loss and cardiopulmonary bypass priming volumes. The relative loss of erythrocyte mass and degree of hemodilution may therefore be greater in women, increasing the probability of reaching an intraoperative Hb concentration at which transfusion is considered [2,3,6,14]. Nevertheless, the optimal preoperative Hb threshold remains controversial and appears to depend on the outcome under consideration. Studies deriving thresholds from RBC transfusion, acute kidney injury, or composite postoperative complications have reported values ranging from those close to the WHO criteria to 13–14 g/dL or higher [6,14,15]. A population-based definition of anemia, an outcome-associated risk threshold, and a therapeutic target for preoperative optimization should therefore not be considered interchangeable concepts.

Available evidence is limited by predominantly single-center cohorts, relatively small sample sizes, heterogeneous cardiac procedures, and variable definitions of anemia. Few large multicenter studies have evaluated the clinical implications of the WHO sex-specific definition compared with a PBM-oriented uniform Hb threshold in patients undergoing elective open cardiac valve surgery (CVS). It also remains uncertain whether women with an Hb concentration of 12.0–12.9 g/dL constitute a clinically relevant but underrecognized risk group in elective open CVS, and whether sex modifies the continuous association between preoperative Hb and RBC transfusion, postoperative complications, hospital length of stay, or mortality. In addition, the association between documented preoperative treatment with intravenous iron and/or erythropoiesis-stimulating agents (ESAs) and subsequent transfusion exposure remains insufficiently characterized in routine multicenter practice.

The primary aim of this study was to assess whether women with a preoperative Hb concentration of 12.0–12.9 g/dL—classified as non-anemic according to WHO criteria but below the PBM-oriented anemia threshold of 13 g/dL—represent an underrecognized group at clinically relevant risk of RBC transfusion after elective open CVS, and whether their transfusion risk differs from that of men with comparable preoperative Hb. Secondary aims were to evaluate the associations of preoperative Hb, modeled as a continuous variable, with postoperative complications, hospital length of stay, and in-hospital mortality within 30 days, and to determine whether these associations differed by sex. Because evidence on sex-specific Hb–outcome associations beyond transfusion is limited and inconsistent [1,2,3], these secondary analyses were considered exploratory. In addition, we performed an exploratory analysis of the association between documented preoperative treatment with intravenous iron and/or an ESA and subsequent RBC transfusion. We hypothesized that women with preoperative Hb concentrations of 12.0–12.9 g/dL would have a higher risk of RBC transfusion than both women with Hb concentrations ≥13.0 g/dL and men within the same Hb stratum, despite being classified as non-anemic according to WHO criteria. For the secondary outcomes, we hypothesized that lower preoperative Hb would be associated with worse postoperative outcomes in both sexes, without an a priori assumption of effect modification by sex.

## 2. Materials and Methods

### 2.1. Study Design and Data Source

We conducted a nationwide multicenter retrospective cohort study using routinely collected data from the Spanish Maturity Assessment Model for Patient Blood Management (MAPBM) registry. The MAPBM project was developed as a structured benchmarking framework to assess institutional PBM maturity, identify areas for improvement, and compare transfusion-related practices and outcomes across participating hospitals [16]. The MAPBM registry integrates perioperative, laboratory, pharmacy, transfusion, surgical, and administrative data from centers with heterogeneous levels of PBM implementation, and participating hospitals have been incorporated progressively since the inception of the project. The study included adults undergoing elective open CVS in 40 Spanish hospitals between January 2016 and December 2024. The cohort partially overlaps with a previously published analysis of the same registry, restricted to 2016–2022, which applied additional exclusion criteria—repeat procedures, aortic surgery, and centers performing fewer than 100 procedures during the study period—and addressed the combined effect of preoperative Hb status and intraoperative blood conservation strategies on transfusion [17]. Of the 20,017 patients in the present analytic cohort, 14,700 (73.4%) met the eligibility criteria of that study; because it included 9889 patients, a maximum of 49.4% of the present cohort could have contributed to both analyses. The present analysis extends the observation period through December 2024, applies different eligibility criteria, and addresses a distinct question based on sex-specific Hb thresholds. The study was reported in accordance with the Strengthening the Reporting of Observational Studies in Epidemiology (STROBE) statement and, given its focus on sex-based differences, with the Sex and Gender Equity in Research (SAGER) guidelines. A completed STROBE checklist is provided as Checklist S5 in the Supplementary Materials.

### 2.2. Ethical Approval

The study was approved by the Clinical Research Ethics Committee of the Hospital del Mar Medical Research Institute, Barcelona, Spain (protocol No. 2021/9924; approved on 22 June 2021), and was conducted in accordance with the Declaration of Helsinki. The same committee waived the requirement for individual informed consent, given the retrospective design and the exclusive use of routinely collected data that were de-identified at source before being transferred to the researchers.

### 2.3. Study Population

Eligible procedures were identified using specific International Classification of Diseases (ICD) codes for elective open CVS. Transcatheter valve procedures were not eligible. We excluded non-elective operations, procedures involving concomitant coronary artery bypass grafting or heart transplantation, patients with endocarditis, and records with unknown surgical complexity. The complete coding algorithm and operational definitions are provided in Supplementary Table S1.

Pre-existing comorbidities were identified from diagnosis codes recorded at admission and classified according to the Charlson comorbidity index using the Quan adaptation [18]; the corresponding ICD codes are provided in Supplementary Table S1. Comorbidity burden was summarized as the number of recorded Charlson comorbidity categories (0, 1–2, 3–4, or ≥5), rather than as the weighted index score. Surgical complexity was classified according to the number of valves operated on as single-, double-, or triple-valve surgery. Valve position was derived from ICD-10-PCS procedure codes, using the fourth character to identify the aortic, mitral, pulmonary, or tricuspid valve. Valve-position categories were not mutually exclusive, as more than one valve could be operated on during the same procedure. Sex was recorded in the MAPBM registry as the administrative sex documented in the hospital electronic health record (female or male); no information on gender identity was available, and the terms “women” and “men” are used throughout to refer to registered sex.

### 2.4. Preoperative Hemoglobin Assessment

The primary exposure was the preoperative Hb concentration, defined as the measurement obtained closest to surgery within the preceding 90 days. This value could therefore reflect Hb after any preoperative treatment administered during that interval. Patients for whom no Hb value obtained within this 90-day window was available in the MAPBM database were excluded from Hb-based analyses. Importantly, the absence of a recorded Hb value in the registry does not imply that preoperative laboratory testing was not performed. Hb was examined both as a continuous variable and using three prespecified categories: <12.0, 12.0–12.9, and ≥13.0 g/dL. These categories allowed comparison of the WHO sex-specific definition of anemia—Hb <12.0 g/dL in women and <13.0 g/dL in men—with a PBM-oriented definition using Hb <13.0 g/dL in both sexes.

As a sensitivity analysis, we used the earliest available Hb measurement within 120 days before surgery. Because this measurement was not required to precede anemia treatment, it cannot necessarily be interpreted as pretreatment Hb.

### 2.5. Preoperative Anemia Treatment

Documented preoperative anemia treatment was defined as any recorded administration of intravenous iron and/or an ESA within 120 days before surgery. For treatment-stratified analyses, patients were classified into four mutually exclusive categories: no documented intravenous iron or ESA, intravenous iron only, ESA only, and intravenous iron plus an ESA. Treatment selection, dose, timing, and monitoring were determined by local institutional protocols and were not standardized across participating centers. Because the primary exposure was the Hb value closest to surgery, patients who responded to treatment may be represented in higher Hb strata; treatment-stratified comparisons are therefore descriptive and should not be interpreted as estimates of treatment effectiveness.

### 2.6. Outcomes

The primary outcome was receipt of at least one unit of allogeneic RBCs from the time of surgery until hospital discharge, encompassing both intraoperative and postoperative transfusions.

Secondary outcomes were all-cause in-hospital mortality within 30 days of the initial hospital admission, including deaths during readmission to the same hospital only if they occurred within this 30-day period; occurrence of at least one postoperative complication; and hospital length of stay. Deaths occurring outside the hospital after discharge were not captured by the registry. Postoperative complications were analyzed as a composite outcome; patients with more than one recorded complication were counted once. Complications were identified from prespecified ICD diagnosis codes recorded during the index admission or during any readmission to the same hospital within 30 days after discharge. Hospital length of stay was defined as the number of days from hospital admission to discharge. The complication definitions and corresponding codes are provided in Supplementary Table S1.

### 2.7. Statistical Analysis

Continuous variables were summarized as mean and standard deviation or median and interquartile range, as appropriate, and categorical variables as counts and percentages. Comparisons between women and men used Student’s *t*-test or the Mann–Whitney U test for continuous variables and the chi-square test or Fisher’s exact test for categorical variables.

Mixed-effects logistic regression was used for RBC transfusion, postoperative complications, and in-hospital mortality within 30 days. Mixed-effects Poisson regression with robust standard errors was used for hospital length of stay. All models were adjusted for age, comorbidity burden, surgical complexity, valve position, and year of surgery and included a random intercept for participating center. For RBC transfusion, preoperative Hb was assessed both categorically (<12.0, 12.0–12.9, and ≥13.0 g/dL) and continuously; secondary outcomes were analyzed with Hb as a continuous variable. Categorical transfusion models included sex, Hb category, and their interaction. The overall sex × Hb category interaction was tested, and adjusted transfusion probabilities and post-estimation contrasts comparing women with men within each Hb stratum were reported as probability differences and adjusted odds ratios (aORs) with 95% confidence intervals. Continuous models included a sex × Hb interaction, and adjusted outcome estimates were plotted across the observed Hb range.

Analyses of preoperative anemia treatment were exploratory. Analyses involving Hb used available cases without imputation. To assess differences in the availability of preoperative Hb measurements, patients with and without an available measurement were matched 1:1 within center using a propensity score based on age, sex, comorbidity burden, surgical complexity, and year of surgery. Sensitivity analyses using the earliest Hb measurement within 120 days before surgery are presented in Supplementary Tables S2 and S3.

Between-center variation in RBC transfusion was quantified using the intraclass correlation coefficient (ICC) and the median odds ratio (MOR) from the mixed-effects logistic regression model. In an exploratory center-level analysis, risk-adjusted transfusion rates were displayed using a funnel plot with 95% and 99.8% control limits. The association between center-level observed-to-expected ratios for RBC transfusion and postoperative complications was assessed using patient-volume-weighted linear regression. This association analysis was restricted to centers with at least 45 patients.

All tests were two-sided, with *p* < 0.05 considered statistically significant; no adjustment was made for multiple comparisons. Analyses were performed using Stata/BE version 18.0 (StataCorp LLC, College Station, TX, USA).

## 3. Results

**Figure 1:**
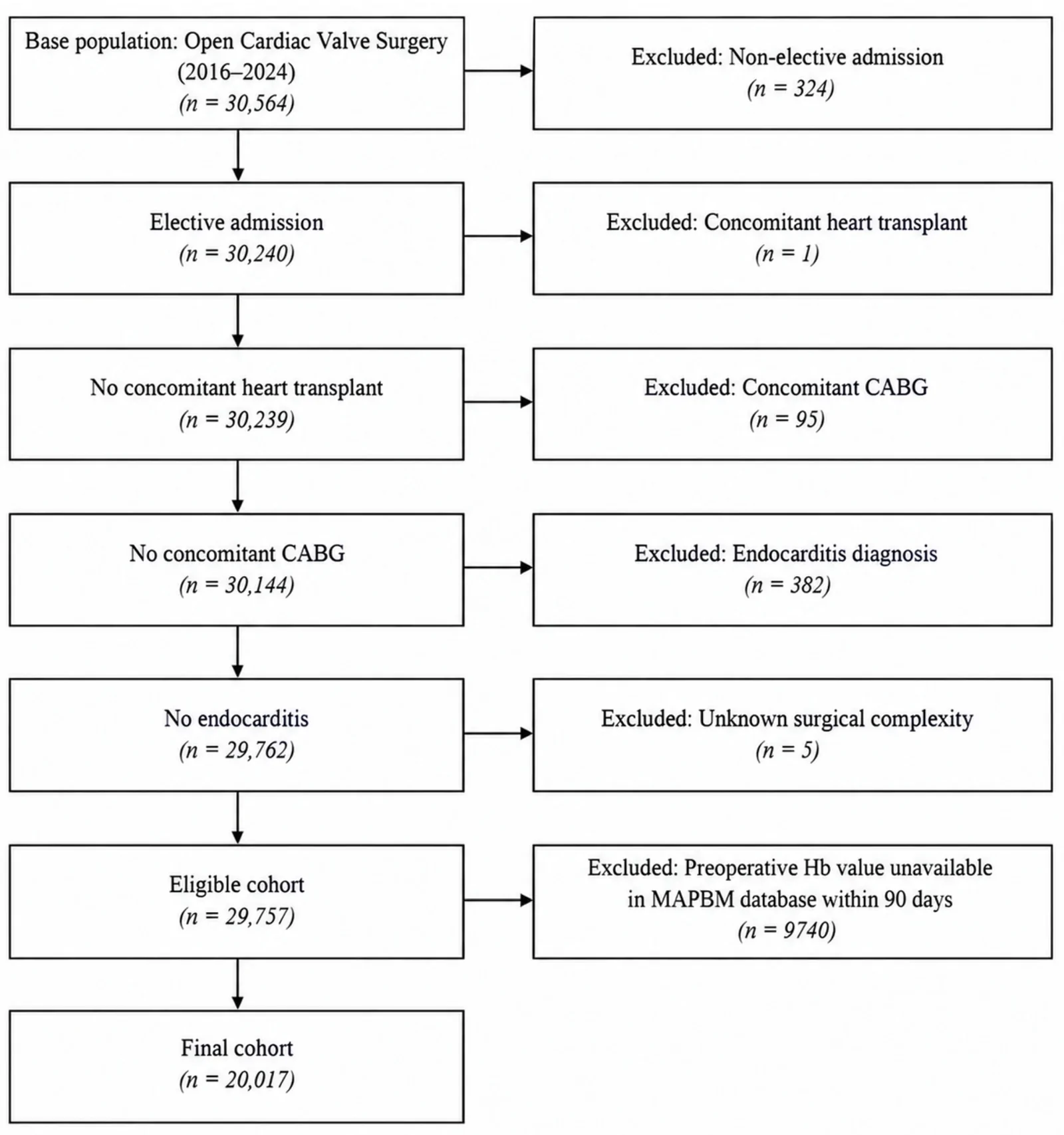
Flow chart of patient selection and exclusion for the study cohort undergoing elective open cardiac valve surgery (CVS). Abbreviations: CABG, coronary artery bypass grafting; CVS, cardiac valve surgery.

A total of 30,564 patients undergoing open CVS in 40 Spanish hospitals between 2016 and 2024 were identified (Figure 1). The source cohort had already been defined by the registry process algorithm, which excludes transcatheter procedures, concomitant coronary artery bypass grafting, heart transplantation, and endocarditis; the exclusions listed below therefore correspond to residual cases identified on secondary review of diagnosis and procedure codes. After excluding 324 patients who underwent non-elective surgery, one patient who underwent concomitant heart transplantation, 95 patients who underwent concomitant coronary artery bypass grafting, 382 patients with endocarditis, and five patients with unknown surgical complexity, 29,757 patients comprised the eligible study cohort. Of these, preoperative Hb values obtained within 90 days before surgery were not available in the MAPBM database for 9740 patients (32.7%), who were therefore excluded from Hb-based analyses. The complete-case cohort for these analyses comprised 20,017 patients from 32 centers. Of the 9740 patients without an available measurement, 1929 were from eight centers that did not report laboratory data to the registry; the remaining 7811 were from the 32 centers contributing laboratory data and were compared with the analyzed cohort before and after propensity-score matching (Supplementary Table S4).

Among the 20,017 patients included in the Hb-based analyses, 9247 (46.2%) were women and 10,770 (53.8%) were men (Table 1). Women were older than men (mean age, 67.6 ± 13.6 vs. 64.3 ± 14.3 years; *p* < 0.001) and had lower preoperative Hb concentrations (12.8 ± 1.5 vs. 13.8 ± 1.9 g/dL; *p* < 0.001). A quarter of women (25.2%) had Hb concentrations of 12.0–12.9 g/dL, more than twice the proportion observed among men (11.8%; *p* < 0.001). Conversely, Hb concentrations ≥13.0 g/dL were more frequent in men than in women (72.2% vs. 49.8%; *p* < 0.001). Women in the 12.0–12.9 g/dL stratum would be classified as non-anemic according to WHO criteria, despite having Hb concentrations below the PBM-oriented threshold of 13.0 g/dL.

Baseline comorbidity profiles also differed by sex. Women more frequently had congestive heart failure and rheumatologic (connective tissue) disease, whereas men had higher prevalences of diabetes mellitus, diabetes with chronic complications, chronic pulmonary disease, renal disease, peripheral vascular disease, previous myocardial infarction, mild liver disease, cancer, metastatic solid tumor, and AIDS/HIV (all *p* < 0.05). Despite their older age, women were more frequently classified in the lower comorbidity-burden categories but had greater surgical complexity, with higher proportions undergoing double- and triple-valve procedures. Valve position also differed by sex. Mitral (47.1% vs. 27.0%) and tricuspid (6.4% vs. 2.7%) valve procedures were more frequent in women, whereas aortic valve procedures were more frequent in men (78.3% vs. 62.0%; all *p* < 0.001).

Unadjusted outcomes also differed by sex (Table 1). Women were more frequently transfused than men (63.4% vs. 39.8%; *p* < 0.001) and received a greater mean number of RBC units (2.22 vs. 1.60; *p* < 0.001). Postoperative complications (41.0% vs. 39.1%; *p* = 0.006), in-hospital mortality within 30 days (3.9% vs. 2.2%; *p* < 0.001), and mean hospital length of stay (14.1 vs. 13.2 days; *p* < 0.001) were also higher in women. These comparisons are unadjusted and do not account for the older age and greater surgical complexity observed in women.

Observed RBC transfusion rates decreased stepwise with increasing preoperative Hb concentrations in both sexes (Table 2). Among women, transfusion occurred in 78.1% of those with Hb <12.0 g/dL, 68.0% of those with Hb 12.0–12.9 g/dL, and 53.7% of those with Hb ≥13.0 g/dL (*p* < 0.001 across strata). Women with Hb concentrations of 12.0–12.9 g/dL therefore had an observed transfusion rate 14.3 percentage points higher than women with Hb ≥13.0 g/dL, despite being classified as non-anemic according to WHO criteria. Women also had higher transfusion rates than men within every Hb stratum: 78.1% versus 69.3% for Hb <12.0 g/dL, 68.0% versus 52.7% for Hb 12.0–12.9 g/dL, and 53.7% versus 31.2% for Hb ≥13.0 g/dL (all *p* < 0.001).

Documented preoperative treatment with intravenous iron and/or an ESA was infrequent overall (7.9% of women and 6.1% of men), and combined treatment with both agents was rare (50 women and 32 men; 0.4% of the cohort). Within the 12.0–12.9 g/dL stratum, treatment was documented less often in women than in men (8.7% vs. 11.9%; *p* = 0.002), whereas in the ≥13.0 g/dL stratum it was documented more often in women (5.8% vs. 3.7%; *p* < 0.001), with no significant difference among patients with Hb <12.0 g/dL (11.4% vs. 12.7%; *p* = 0.220).

After multivariable adjustment, women had a significantly higher probability of RBC transfusion than men within each preoperative Hb stratum (Table 3). The adjusted absolute difference in transfusion probability between women and men was 10.4 percentage points (95% CI, 6.9–13.9) for Hb <12.0 g/dL, 15.1 percentage points (95% CI, 11.4–18.9) for Hb 12.0–12.9 g/dL, and 20.6 percentage points (95% CI, 17.7–23.5) for Hb ≥13.0 g/dL. Corresponding aORs for women versus men were 1.63 (95% CI, 1.40–1.89), 1.85 (95% CI, 1.59–2.16), and 2.59 (95% CI, 2.38–2.82), respectively (all *p* < 0.001). The overall interaction between sex and categorical Hb strata was statistically significant (*p* < 0.001), indicating heterogeneity in the adjusted between-sex transfusion differences across Hb categories. Between-center variability was substantial, with an intraclass correlation coefficient of 0.167 and a median odds ratio of 2.168. In an exploratory center-level analysis restricted to 28 centers with at least 45 patients, there was no significant association between risk-adjusted RBC transfusion and postoperative complication ratios (patient-volume-weighted slope, −0.04; 95% CI, −0.29 to 0.21; *p* = 0.763; Supplementary Figure S1). In sensitivity analyses using the earliest available Hb measurement within 120 days before surgery, adjusted sex differences were similar (aORs 1.49, 1.81, and 2.53 for Hb < 12.0, 12.0–12.9, and ≥13.0 g/dL, respectively; Supplementary Table S3).

**Figure 2:**
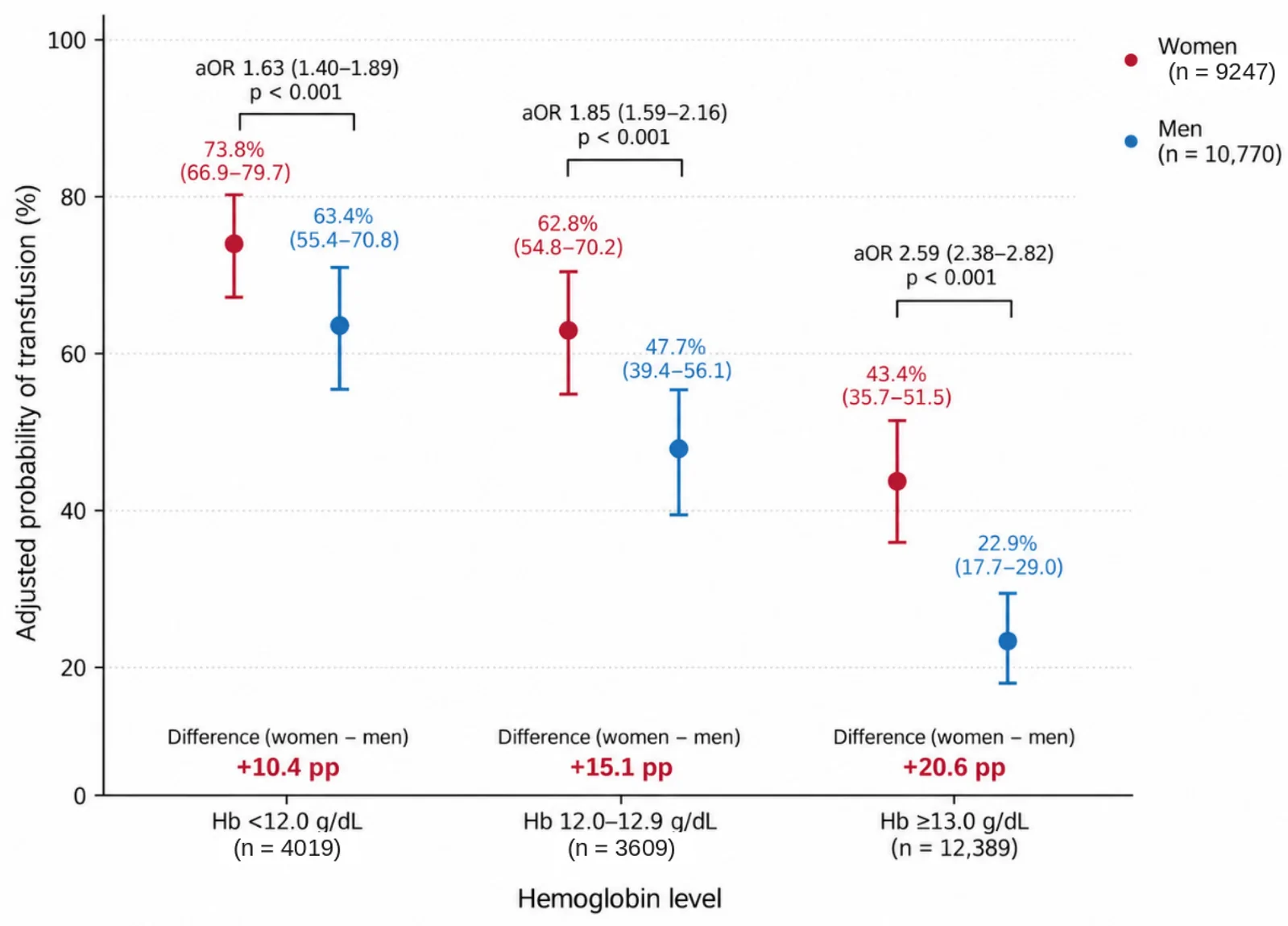
Adjusted probability of RBC transfusion according to sex and preoperative Hb stratum. Points represent model-based adjusted probabilities, and error bars represent 95% CIs, estimated using a mixed-effects logistic regression model including sex, Hb category, and their interaction, adjusted for age, comorbidity burden, surgical complexity, valve position, and year of surgery, with a random intercept for participating center. Brackets show the adjusted comparison between women and men within each Hb stratum (aOR, 95% CI); adjusted probability differences (women minus men, in percentage points) are shown below each stratum. Abbreviations: aOR, adjusted odds ratio; CI, confidence interval; Hb, hemoglobin; RBC, red blood cell.

The model-based adjusted transfusion probabilities across the six combined sex–Hb categories are shown in Figure 2. Women had higher adjusted probabilities of RBC transfusion than men within each Hb stratum. Of particular note, the adjusted probability among women with Hb 12.0–12.9 g/dL was nearly identical to that observed among men with Hb <12.0 g/dL (62.8% vs. 63.4%, respectively). This comparison is descriptive and was not intended as a formal test of equivalence.

**Figure 3:**
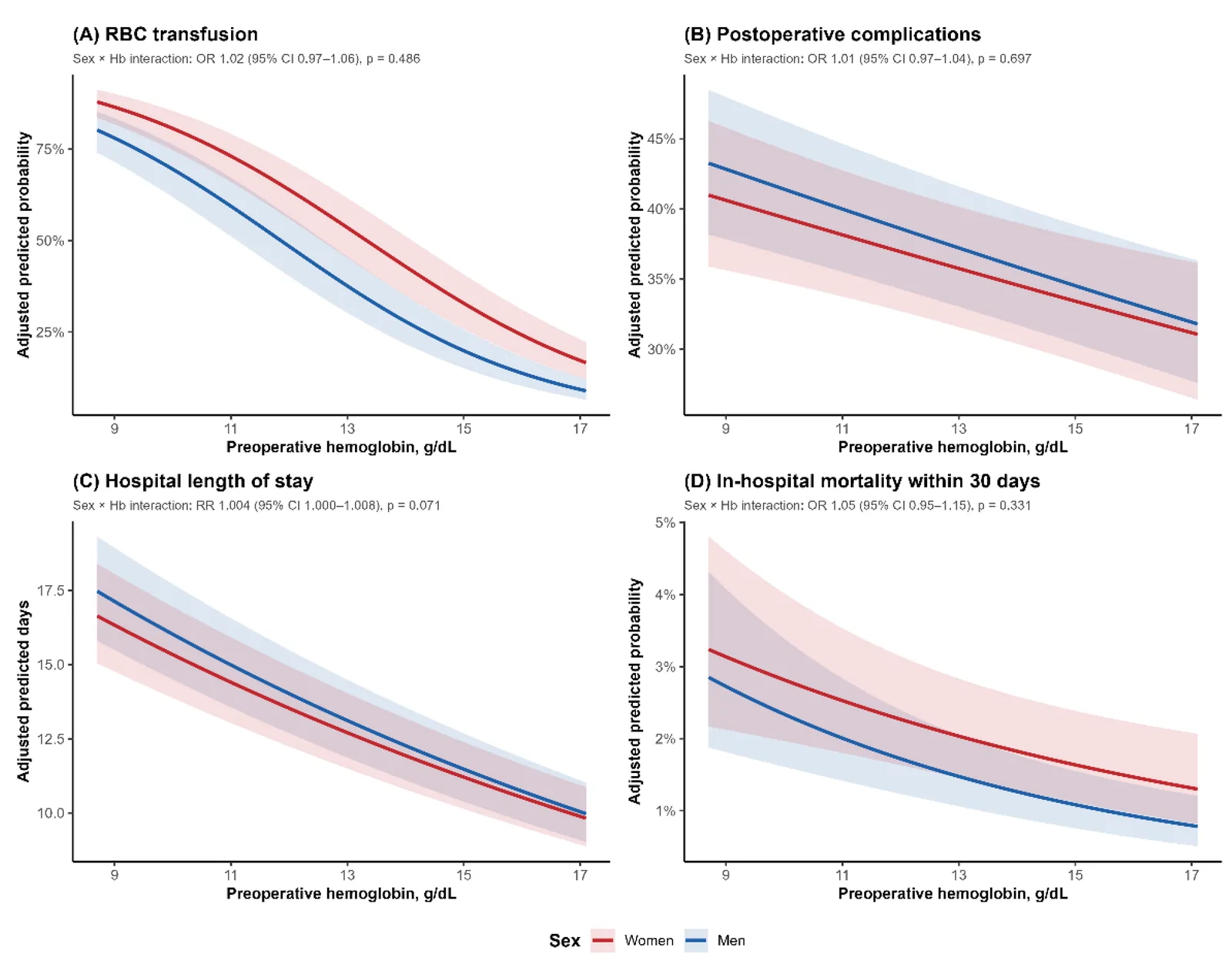
Adjusted associations between preoperative Hb and postoperative outcomes, stratified by sex. (**A**) RBC transfusion; (**B**) postoperative complications; (**C**) hospital length of stay; (**D**) in-hospital mortality within 30 days. Panels (**A**,**B**,**D**) were modeled using mixed-effects logistic regression, and panel C using mixed-effects Poisson regression. All models were adjusted for age, comorbidity burden, surgical complexity, valve position, and year of surgery and included a random intercept for participating center. Shaded areas represent 95% CIs. Abbreviations: CI, confidence interval; Hb, hemoglobin; RBC, red blood cell.

To complement the categorical analyses and assess whether these associations were consistent across the full Hb range, preoperative Hb was subsequently modeled as a continuous variable. Higher preoperative Hb was associated with a progressively lower adjusted probability of RBC transfusion in both women and men (Figure 3). Women maintained a higher predicted probability of transfusion across most of the Hb range; however, there was no statistically significant evidence that the association between Hb and RBC transfusion differed by sex (sex × Hb interaction: OR 1.02, 95% CI 0.97–1.06; *p* = 0.486) (Table 4).

Similar inverse associations with preoperative Hb were observed for postoperative complications and in-hospital mortality within 30 days (Figure 3). The adjusted probability of postoperative complications decreased with increasing Hb in both sexes, with largely overlapping trajectories and no statistically significant sex × Hb interaction (OR 1.01, 95% CI 0.97–1.04; *p* = 0.697) (Table 4). Likewise, the adjusted probability of in-hospital mortality within 30 days declined as preoperative Hb increased, with no significant evidence of effect modification by sex (OR 1.05, 95% CI 0.95–1.15; *p* = 0.331) (Table 4).

Adjusted mean hospital length of stay also decreased progressively with increasing preoperative Hb in both sexes (Figure 3). The sex × Hb interaction was not statistically significant (RR 1.004, 95% CI 1.000–1.008; *p* = 0.071), providing no clear evidence that the association between Hb and hospital length of stay differed by sex (Table 4). However, the magnitude of this interaction was small, and predicted mean length of stay remained broadly comparable between women and men across most of the Hb range.

## 4. Discussion

In this nationwide multicenter cohort of patients undergoing elective open CVS, lower preoperative Hb was associated with greater RBC transfusion risk and worse postoperative outcomes in both sexes. Unadjusted rates of transfusion, postoperative complications, in-hospital mortality within 30 days, and hospital length of stay were all higher in women, although women were also older and underwent more complex procedures. Women had a higher adjusted probability of RBC transfusion than men within each Hb stratum and across most of the continuous Hb range, including the 12.0–12.9 g/dL range classified as non-anemic according to WHO criteria. The sex difference was larger at higher Hb strata in the categorical model, but the sex × Hb interaction was not significant when Hb was modeled continuously. Thus, the main finding is not a disproportionately stronger effect of declining Hb in women, but a persistently higher transfusion probability at comparable Hb concentrations.

The clinical relevance of the 12.0–12.9 g/dL range in women is supported by previous studies. Blaudszun et al. [11] reported higher transfusion rates and longer hospital stays in women with “borderline anemia” than in those with Hb ≥13 g/dL and Ștefan et al. [12] recently confirmed a stepwise increase in perioperative transfusion risk across decreasing Hb strata in women undergoing cardiac surgery. Similar associations with transfusion exposure and selected postoperative complications have been reported in women undergoing other major surgical procedures [13]. Our findings extend these observations to a large multicenter valve-surgery cohort and show that women with Hb 12.0–12.9 g/dL also have a higher adjusted probability of RBC transfusion than men within the same Hb stratum.

An additional finding was that the sex difference in transfusion risk did not disappear at higher Hb concentrations. The adjusted absolute difference between women and men was greatest among patients with Hb ≥13.0 g/dL, and the overall sex × Hb category interaction was statistically significant. However, this category has no upper bound, and mean Hb within it was 0.75 g/dL lower in women than in men (13.99 vs. 14.74 g/dL); part of the larger difference in this stratum may reflect differences in Hb within this broad category, consistent with the absence of a significant sex × Hb interaction when Hb was modeled continuously. By contrast, mean Hb was virtually identical in women and men within the 12.0–12.9 g/dL stratum (12.47 vs. 12.49 g/dL), so the adjusted 15.1-percentage-point difference observed in this range is unlikely to be explained solely by differences in mean Hb within the stratum, and below 12.0 g/dL women had a slightly higher mean Hb than men. Taken together, these findings suggest that the excess transfusion risk associated with female sex persists across the Hb range, consistent with sensitivity analyses using the earliest available Hb measurement and with previous cardiac surgical studies identifying female sex as an independent predictor of RBC transfusion [1,2,14].

Our findings support Hb < 13.0 g/dL as a pragmatic threshold for recognizing perioperative risk in both sexes, not as a biological cutoff or a proven therapeutic target. Outcome-derived thresholds have varied considerably across studies. Ripoll et al. identified sex-specific spline knots at approximately 13 g/dL in women and 14 g/dL in men [6], whereas Cavalli et al. estimated that women required a preoperative Hb of approximately 13.3 g/dL to achieve a transfusion probability comparable with that of men at a lower Hb concentration [14]. By contrast, Kattou et al. identified a threshold of approximately 11.8 g/dL in women for postoperative complications [15], and different values have been reported in orthopedic and mixed surgical populations [19,20,21]. These discrepancies likely reflect differences in surgical setting, outcome definition, and analytical approach. More recently, a large surgical cohort showed that sex-related differences in the association between preoperative anemia and postoperative outcomes varied according to the endpoint considered [22]. A diagnostic definition, a risk-discrimination threshold, and a therapeutic target should therefore not be considered interchangeable. In our cohort, the evidence was strongest for transfusion-risk identification; sex did not significantly modify the continuous association of Hb with postoperative complications or in-hospital mortality within 30 days. Accordingly, these data do not support deriving a sex-specific mortality threshold.

The greater transfusion probability observed in women at comparable Hb concentrations may reflect perioperative factors not captured in the registry. At a given Hb concentration, women generally have lower absolute circulating blood volume and red-cell mass and may therefore experience greater relative hemodilution during cardiopulmonary bypass [2,14]. Intraoperative anemia has also been shown to mediate a substantial proportion of the excess operative mortality observed in women undergoing coronary artery bypass grafting [3]. However, the observed sex difference could also reflect variation in transfusion decision-making, because transfusion triggers and clinical indications were not available in the registry. Distinguishing biological vulnerability from practice-related differences would require information on body size, estimated blood volume, bypass priming volume, nadir intraoperative Hb, cardiopulmonary bypass duration, perioperative blood loss, and documented transfusion triggers. The relationship between anemia, transfusion, and postoperative outcomes may also differ according to the endpoint. Warner et al. found that intraoperative transfusion accounted for relatively little of the association between preoperative anemia and acute kidney injury but explained a greater proportion of the association with hospital length of stay [7]. Because transfusion occurs after the preoperative Hb exposure and may mediate part of its association with postoperative outcomes, dedicated mediation methods are required to distinguish direct from transfusion-mediated associations appropriately [23]. Exploratory center-level analyses identified substantial between-center variability in transfusion (MOR 2.168), without a significant association between adjusted transfusion and postoperative complication ratios (Supplementary Figure S1). Given the ecological design, this finding cannot establish the absence of patient-level associations or causal effects.

The treatment-stratified analyses should be interpreted cautiously. Documented preoperative treatment was less frequent in women than in men within the 12.0–12.9 g/dL stratum despite their higher transfusion risk, but this observation cannot establish differences in access to or allocation of treatment. It may reflect heterogeneity between centers in screening pathways, treatment eligibility, timing of evaluation, and local Hb thresholds. In addition, intravenous iron and ESAs were administered according to clinical practice rather than randomized allocation, creating substantial potential for confounding by indication. The preoperative Hb used in the primary analysis may therefore represent a treatment-modified concentration rather than untreated baseline Hb. Furthermore, patients with similar Hb concentrations may differ in underlying iron status and previous treatment exposure. Information on anemia etiology, iron indices, treatment dose, adherence, and hematologic response was unavailable. Although treatment administration dates were recorded, their temporal relationship to the selected Hb measurement was not examined. ESA-treated groups were small. Evidence from cardiac surgery nevertheless suggests that treatment of anemia or iron deficiency can increase Hb and reduce RBC transfusion, although benefits for major clinical outcomes remain less consistently demonstrated [24,25,26,27,28]. Our findings therefore neither establish comparative treatment effectiveness nor justify treating all women with Hb 12.0–12.9 g/dL with iron or ESAs. Rather, Hb <13.0 g/dL should prompt timely etiologic evaluation and individualized management within a multimodal PBM pathway, consistent with contemporary perioperative and cardiac-surgery recommendations [8,28,29,30]. Such an approach integrates anemia management with strategies to minimize blood loss and avoid unnecessary transfusion rather than relying on a single pharmacological intervention or Hb target [17,31,32].

From an equity perspective, one in four women with an available preoperative Hb measurement (2333/9247; 25.2%) had concentrations of 12.0–12.9 g/dL and would therefore be classified as non-anemic according to WHO criteria despite a clinically relevant transfusion risk. In perioperative pathways in which anemia definitions determine further investigation or treatment, exclusive reliance on sex-specific population thresholds could therefore lead to under-recognition of risk in a substantial proportion of women. This does not imply that the WHO definition itself causes the observed sex disparity, nor that adopting a uniform threshold would eliminate it. Rather, our findings suggest that population-based anemia definitions should not automatically be used as perioperative risk thresholds or as the sole criterion for PBM evaluation [2,12,28].

The principal strengths of this study are its large nationwide multicenter cohort, substantial representation of women, contemporary study period, and specific focus on elective open CVS. The complementary categorical and continuous analyses allowed clinically familiar Hb strata to be interpreted alongside adjusted outcome trajectories and formal sex × Hb interactions, while multivariable models accounted for age, comorbidity burden, surgical complexity, valve position, year of surgery, and center-level clustering. Several limitations should nevertheless be acknowledged. The retrospective observational design precludes causal inference and remains susceptible to residual confounding. Preoperative Hb values were not available in the MAPBM database for 9740 of 29,757 otherwise eligible patients (32.7%). This reflects limitations in laboratory data availability rather than necessarily indicating that preoperative blood testing was not performed. Potential explanations include preoperative laboratory tests performed at referring hospitals or in primary care, whose results may have been available to clinicians but were not included in the laboratory data extracted by MAPBM, as well as incomplete laboratory data extraction or reporting across participating centers and years. Nevertheless, the exclusion of these patients may introduce selection bias. Although patients with and without an available Hb value had similar baseline characteristics and, after propensity-score matching, similar rates of postoperative complications and in-hospital mortality within 30 days, those without an available Hb value were less frequently transfused (33.4% vs. 54.0%) and had shorter hospital stays (Supplementary Table S4). In addition, the last value obtained within 90 days may not always represent Hb immediately before surgery. Moreover, in patients receiving preoperative anemia treatment, this measurement may reflect treatment-modified Hb rather than untreated baseline Hb. Our primary analyses therefore assess the prognostic association between the Hb value recorded closest to surgery and transfusion risk, rather than the causal effect of anemia treatment. The registry did not capture iron status, anemia etiology, or the intraoperative and hemodilution-related variables noted above. Surgical complexity was characterized by the number of valves operated on, and valve position was included in the adjusted models. However, detailed procedural characteristics, including valve repair versus replacement, reoperative surgery, and cardiopulmonary bypass duration, were not available. Residual confounding related to procedural differences between women and men therefore remains possible. Risk adjustment was based on age, comorbidity burden, surgical complexity, valve position, year of surgery, and center; validated cardiac surgical risk scores such as EuroSCORE II or the Society of Thoracic Surgeons score were not available in the registry, and residual confounding by baseline surgical risk cannot be excluded. Comorbidities and postoperative complications were derived from administrative diagnosis codes and may therefore be subject to misclassification. Mortality was ascertained as in-hospital death within 30 days of admission, including readmissions to the same hospital; deaths occurring after discharge outside the hospital were not captured, which may have underestimated mortality. Treatment exposure lacked detailed information on indication, dose, and hematologic response. Although treatment administration dates were available, the temporal relationship between treatment and the selected Hb measurement was not evaluated. The cohort comprised Spanish hospitals participating in a national PBM benchmarking program, and anemia management pathways and transfusion practice may differ in other settings, limiting generalizability. Finally, the predefined Hb strata should be interpreted as clinically useful risk categories rather than validated biological cutoffs or therapeutic targets.

## 5. Conclusions

In patients undergoing elective open CVS, women had a higher probability of RBC transfusion than men at comparable preoperative Hb concentrations, including the 12.0–12.9 g/dL range classified as non-anemic according to the WHO criteria. Lower Hb was associated with increasing transfusion risk, more postoperative complications, longer hospital stay, and higher in-hospital mortality within 30 days in both sexes; although sex differences varied across categorical Hb strata, the continuous sex × Hb interaction was not statistically significant, and sex did not significantly modify the associations of Hb with postoperative complications or in-hospital mortality within 30 days. These findings support considering Hb < 13.0 g/dL as a pragmatic, sex-inclusive threshold for perioperative risk recognition and etiologic evaluation, rather than as a universal therapeutic target. Prospective studies incorporating blood volume, cardiopulmonary bypass-related hemodilution, perioperative bleeding, transfusion triggers, and treatment response are needed to determine whether such an approach can reduce transfusion exposure and improve perioperative equity.

## Figures and Tables

**Table 1:** Baseline characteristics and unadjusted outcomes of patients with available preoperative Hb measurements, stratified by sex.

*p*-Value	Men (*n* = 10,770)	Women (*n* = 9247)	Overall (*N* = 20,017)	Characteristic
0.049				**Year of surgery, n (%)**
	936 (8.7)	837 (9.1)	1773 (8.9)	2016
	1363 (12.7)	1158 (12.5)	2521 (12.6)	2017
	1418 (13.2)	1269 (13.7)	2687 (13.4)	2018
	1424 (13.2)	1197 (12.9)	2621 (13.1)	2019
	1018 (9.5)	799 (8.6)	1817 (9.1)	2020
	1023 (9.5)	849 (9.2)	1872 (9.4)	2021
	1235 (11.5)	1188 (12.8)	2423 (12.1)	2022
	1126 (10.5)	939 (10.2)	2065 (10.3)	2023
	1227 (11.4)	1011 (10.9)	2238 (11.2)	2024
<0.001	64.3 (14.3)	67.6 (13.6)	65.8 (14.1)	**Age, years, mean (SD)**
<0.001	13.8 (1.9)	12.8 (1.5)	13.3 (1.8)	**Preoperative Hb within 90 days before surgery, g/dL, mean (SD)** †
<0.001				**Hemoglobin stratum, n (%)**
	1713 (15.9)	2306 (24.9)	4019 (20.1)	<12.0 g/dL
	1276 (11.8)	2333 (25.2)	3609 (18.0)	12.0–12.9 g/dL
	7781 (72.2)	4608 (49.8)	12,389 (61.9)	≥13.0 g/dL
				**Mean Hb within stratum, g/dL, mean (SD)**
<0.001	10.59 (1.02)	10.77 (0.97)	10.70 (1.00)	<12.0 g/dL
0.256	12.49 (0.30)	12.47 (0.29)	12.48 (0.29)	12.0–12.9 g/dL
<0.001	14.74 (1.10)	13.99 (0.83)	14.46 (1.07)	≥13.0 g/dL
<0.001				**Comorbidity burden, *n* (%)**
	4391 (40.8)	4347 (47.0)	8738 (43.7)	0 comorbidities
	5498 (51.0)	4399 (47.6)	9897 (49.4)	1–2 comorbidities
	827 (7.7)	486 (5.3)	1313 (6.6)	3–4 comorbidities
	54 (0.5)	15 (0.2)	69 (0.3)	≥5 comorbidities
				**Comorbidities, *n* (%)**
0.004	1505 (14.0)	1426 (15.4)	2931 (14.6)	Congestive heart failure
0.047	2039 (18.9)	1649 (17.8)	3688 (18.4)	Diabetes mellitus
<0.001	249 (2.3)	144 (1.6)	393 (2.0)	Diabetes with chronic complications
<0.001	1476 (13.7)	1032 (11.2)	2508 (12.5)	Chronic pulmonary disease
0.001	1034 (9.6)	764 (8.3)	1798 (9.0)	Renal disease
<0.001	1830 (17.0)	712 (7.7)	2542 (12.7)	Peripheral vascular disease
0.798	536 (5.0)	452 (4.9)	988 (4.9)	Cerebrovascular disease
<0.001	487 (4.5)	227 (2.5)	714 (3.6)	Myocardial infarction history
0.007	413 (3.8)	289 (3.1)	702 (3.5)	Mild liver disease
0.387	49 (0.5)	51 (0.6)	100 (0.5)	Moderate or severe liver disease
<0.001	130 (1.2)	275 (3.0)	405 (2.0)	Rheumatologic (connective tissue) disease
<0.001	228 (2.1)	118 (1.3)	346 (1.7)	Cancer history
0.009	29 (0.3)	9 (0.1)	38 (0.2)	Metastatic solid tumor
0.505	22 (0.2)	24 (0.3)	46 (0.2)	Dementia
0.891	43 (0.4)	39 (0.4)	82 (0.4)	Peptic ulcer disease
0.424	79 (0.7)	78 (0.8)	157 (0.8)	Hemiplegia or paraplegia
0.046	17 (0.2)	5 (0.1)	22 (0.1)	AIDS/HIV
<0.001				**Surgical complexity, *n* (%)**
	9783 (90.8)	7762 (83.9)	17,545 (87.7)	Single-valve procedure
	959 (8.9)	1401 (15.2)	2360 (11.8)	Double-valve procedure
	28 (0.3)	84 (0.9)	112 (0.6)	Triple-valve procedure
				**Valve operated on, *n* (%)** ‡
<0.001	8429 (78.3)	5734 (62.0)	14,163 (70.8)	Aortic
<0.001	2904 (27.0)	4353 (47.1)	7257 (36.3)	Mitral
<0.001	287 (2.7)	591 (6.4)	878 (4.4)	Tricuspid
0.864	165 (1.5)	138 (1.5)	303 (1.5)	Pulmonary
				**Outcomes**
<0.001	4285 (39.8)	5863 (63.4)	10,148 (50.7)	RBC transfusion, *n* (%)
<0.001	1.60 (3.63)	2.22 (3.74)	1.89 (3.69)	RBC units transfused, mean (SD)
0.006	4209 (39.1)	3793 (41.0)	8002 (40.0)	Any postoperative complication, *n* (%)
<0.001	234 (2.2)	357 (3.9)	591 (3.0)	In-hospital mortality within 30 days, *n* (%)
<0.001	13.2 (12.2)	14.1 (12.4)	13.6 (12.3)	Hospital length of stay, days, mean (SD)

† Preoperative Hb was defined as the last available hemoglobin measurement obtained within 90 days before surgery. Data are presented as mean (SD) for continuous variables and *n* (%) for categorical variables. Percentages were calculated using the corresponding column denominators. ‡ Valve-position categories were not mutually exclusive, as patients undergoing multiple-valve surgery could be included in more than one category. Continuous variables were compared using Student’s *t*-test or the Mann–Whitney U test, and categorical variables using the chi-square test or Fisher’s exact test, as appropriate. For categorical variables with more than two levels, the *p*-value shown in the heading row corresponds to the overall distribution across categories. Given the large sample size, small absolute differences may reach statistical significance. Abbreviations: Hb, hemoglobin; RBC, red blood cell; SD, standard deviation.

**Table 2:** RBC transfusion rates according to preoperative Hb stratum, documented preoperative treatment, and sex.

*p*-Value	Men, RBC Transfusion, *n* (%)	Men, *n*	Women, RBC Transfusion, *n* (%)	Women, *n*	Hemoglobin Stratum and Treatment
					**<12.0 g/dL**
<0.001	1001 (67.0)	1495	1582 (77.4)	2043	No documented intravenous iron or ESA
0.797	145 (84.3)	172	180 (83.3)	216	Intravenous iron only
1.000	25 (89.3)	28	21 (91.3)	23	ESA only
0.431	16 (88.9)	18	18 (75.0)	24	Intravenous iron + ESA
**<0.001**	**1187 (69.3)**	**1713**	**1801 (78.1)**	**2306**	**Subtotal**
					**12.0–12.9 g/dL**
<0.001	589 (52.4)	1124	1452 (68.1)	2131	No documented intravenous iron or ESA
0.023	73 (54.1)	135	122 (66.7)	183	Intravenous iron only
1.000	6 (85.7)	7	4 (80.0)	5	ESA only
0.408	4 (40.0)	10	9 (64.3)	14	Intravenous iron + ESA
**<0.001**	**672 (52.7)**	**1276**	**1587 (68.0)**	**2333**	**Subtotal**
					**≥13.0 g/dL**
<0.001	2350 (31.4)	7496	2332 (53.7)	4340	No documented intravenous iron or ESA
<0.001	72 (26.3)	274	137 (54.4)	252	Intravenous iron only
0.545	3 (42.9)	7	3 (75.0)	4	ESA only
1.000	1 (25.0)	4	3 (25.0)	12	Intravenous iron + ESA
**<0.001**	**2426 (31.2)**	**7781**	**2475 (53.7)**	**4608**	**Subtotal**

Data are presented as *n* (%). Hb strata were based on the last available preoperative Hb measurement obtained within 90 days before surgery. Documented preoperative treatment was defined as any recorded administration of intravenous iron and/or an ESA within 120 days before surgery, classified into four mutually exclusive categories. *p*-values compare RBC transfusion rates between women and men within each Hb stratum and treatment subgroup, and were calculated using the chi-square test or Fisher’s exact test, as appropriate. Any documented preoperative treatment was recorded in 733 of 9247 women (7.9%) and 655 of 10,770 men (6.1%). Treatment indication, dose, and hematologic response were unavailable. Although treatment administration dates were recorded, their temporal relationship to the selected Hb measurement was not assessed. Several subgroups contained few patients; treatment-stratified analyses should therefore be interpreted as exploratory. Abbreviations: ESA, erythropoiesis-stimulating agent; Hb, hemoglobin; RBC, red blood cell.

**Table 3:** Adjusted sex differences in RBC transfusion within preoperative hemoglobin strata.

*p*-Value	aOR (95% CI)	Adjusted Probability Difference, Percentage Points (95% CI)	Preoperative Hb Stratum
<0.001	1.63 (1.40–1.89)	10.4 (6.9–13.9)	**<12.0 g/dL**
<0.001	1.85 (1.59–2.16)	15.1 (11.4–18.9)	**12.0–12.9 g/dL**
<0.001	2.59 (2.38–2.82)	20.6 (17.7–23.5)	**≥13.0 g/dL**

All estimates compare women with men within the same preoperative Hb stratum and were derived from a single mixed-effects logistic regression model including sex, Hb category, and their interaction, adjusted for age, comorbidity burden, surgical complexity, valve position, and year of surgery, with a random intercept for participating center. Adjusted probability differences are expressed in percentage points; positive values indicate a higher adjusted probability of RBC transfusion in women. Preoperative Hb was defined as the last available measurement obtained within 90 days before surgery. Abbreviations: aOR, adjusted odds ratio; CI, confidence interval; Hb, hemoglobin; RBC, red blood cell.

**Table 4:** Sex × preoperative Hb interaction in continuous Hb models.

*p*-Value	Sex × Hb Interaction (95% CI)	Model	Outcome
0.486	OR 1.02 (0.97–1.06)	Mixed-effects logistic regression	RBC transfusion
0.697	OR 1.01 (0.97–1.04)	Mixed-effects logistic regression	Postoperative complications
0.071	RR 1.004 (1.000–1.008)	Mixed-effects Poisson regression	Hospital length of stay
0.331	OR 1.05 (0.95–1.15)	Mixed-effects logistic regression	In-hospital mortality within 30 days

All models examined preoperative Hb as a continuous variable and were adjusted for age, comorbidity burden, surgical complexity, valve position, and year of surgery, with a random intercept for participating center. Interaction estimates represent the multiplicative difference in the Hb–outcome association between women and men for each 1 g/dL increase in preoperative Hb. Abbreviations: CI, confidence interval; Hb, hemoglobin; OR, odds ratio; RBC, red blood cell; RR, rate ratio.

## Data Availability

The datasets presented in this article are not readily available because they are subject to institutional, ethical, and data-protection restrictions. Requests to access the datasets should be directed to the corresponding author and are subject to permission from the MAPBM steering committee.

## Supplementary Materials

The following supporting information can be downloaded at: https://www.mdpi.com/article/10.3390/jcm15218632/s1. Table S1: International Classification of Diseases codes and operational definitions used to identify eligible procedures, exclusion criteria, comorbidities, and postoperative complications; Table S2: Sensitivity analysis of RBC transfusion rates according to the earliest available preoperative hemoglobin measurement within 120 days before surgery, documented preoperative treatment, and sex; Table S3: Sensitivity analysis: adjusted sex differences in RBC transfusion within preoperative hemoglobin strata, using the earliest available hemoglobin measurement within 120 days before surgery; Table S4: Patients with and without an available preoperative hemoglobin measurement, before and after propensity-score matching; Supplementary Figure S1: Between-center variability in red blood cell transfusion and postoperative complications; Checklist S5: STROBE Statement—checklist of items that should be included in reports of observational studies.
