# Supplementary material for: When “Normal” Hemoglobin May Not Be Enough: Sex-Specific Anemia Thresholds and Outcomes in Cardiac Surgery
Source: J Clin Med. 2026 Nov 5;15(21):8632. doi: 10.3390/jcm15218632 (PMC13764054; doi:10.3390/jcm15218632)
Supplement: Supplementary file 1 [file jcm-15-08632-s001.zip › jcm-4612235-supplementary.pdf]

Supplementary Material

Table S1. International Classification of Diseases codes and operational definitions used to identify eligible procedures, exclusion criteria, comorbidities, and postoperative complications.

A. Cohort selection and exclusion criteria

Component	Coding system	Code(s)	Operational definition
Eligible elective open cardiac valve surgery	ICD-10-PCS	02QF0ZJ, 02QF0ZZ, 02QG0ZE, 02QG0ZZ, 02QH0ZZ, 02QJ0ZG, 02QJ0ZZ, 02RF07Z, 02RF08Z, 02RF0JZ, 02RF0KZ, 02RG07Z, 02RG08Z, 02RG0JZ, 02RG0KZ, 02RH07Z, 02RH08Z, 02RH0JZ, 02RH0KZ, 02RJ07Z, 02RJ08Z, 02RJ0JZ, 02RJ0KZ	Scheduled admission with an open surgical repair or replacement of the aortic, mitral, pulmonary, or tricuspid valve.
Legacy valve-procedure mapping supplied in the MAPBM dictionary	ICD-9-CM	35.06, 35.08, 35.09, 35.10, 35.11, 35.12, 35.13, 35.14, 35.20, 35.21, 35.22, 35.23, 35.24, 35.25, 35.26, 35.27, 35.28	Parallel legacy mapping reproduced from the MAPBM cardiac-valve selection workbook; transcatheter valve procedures were not included.
Transcatheter valve procedures (TAVI/TAVR)	Cohort eligibility rule	Not applicable	Not eligible. The cohort was restricted to valve procedures performed through an open surgical approach.
Concomitant heart transplantation	ICD-10-PCS	02YA0Z0, 02YA0Z1, 02YA0Z2	Excluded when a heart-transplant procedure was recorded during the same episode.
Concomitant heart transplantation	ICD-9-CM	33.6, 37.51	Excluded when a heart or combined heart-lung transplant procedure was recorded during the same episode.
Concomitant coronary artery bypass grafting	ICD-10-PCS	0210*, 0211*, 0212*, 0213*	Excluded when any MAPBM-listed coronary artery bypass procedure code was recorded during the same episode.
Concomitant coronary artery bypass grafting	ICD-9-CM	36.10–36.17, 36.19	Excluded when a coronary bypass procedure was recorded during the same episode.
Endocarditis	ICD-10-CM	I33, I33.0, I33.9	Excluded when acute or subacute endocarditis was coded during the index episode.
Non-elective surgery	Administrative admission variable	Scheduled/elective admission required	Episodes not classified as scheduled/elective were excluded.
Unknown surgical complexity	Operational definition	Not code-based	Records in which the number of valves operated could not be classified as single-, double-, or triple-valve surgery were excluded from the definitive cohort.

B. Operational definitions

Variable	Operational definition
Preoperative Hb (primary exposure)	Last available hemoglobin measurement obtained within 90 days before surgery.
Sensitivity-analysis Hb	Earliest available hemoglobin measurement obtained within 120 days before surgery.
Hb strata	<12.0 g/dL; 12.0–12.9 g/dL; ≥13.0 g/dL.
Comorbidity burden	Number of Charlson comorbidity categories recorded at admission, grouped as 0, 1–2, 3–4, or ≥5.
Surgical complexity	Single-, double-, or triple-valve surgery according to the number of valves operated.
RBC transfusion	Receipt of at least one unit of allogeneic RBCs from surgery until hospital discharge.
In-hospital mortality within 30 days	All-cause death occurring in the hospital within 30 days of the initial admission, including deaths during readmission to the same hospital only if they occurred within that 30-day period. Deaths occurring outside the hospital were not captured.
Postoperative complication	Occurrence of at least one prespecified postoperative complication identified from diagnosis codes recorded during the index admission or during any readmission to the same hospital within 30 days after discharge.
Documented preoperative anemia treatment	Recorded treatment within 120 days before surgery, classified into four mutually exclusive categories: no documented intravenous iron or ESA, intravenous iron only, ESA only, and intravenous iron plus an ESA.

Variable	Operational definition
Valve position	Aortic, mitral, pulmonary, or tricuspid valve involvement identified from the fourth character of ICD-10-PCS procedure codes. Categories were not mutually exclusive.

C. Comorbidities

Pre-existing comorbidities were classified according to the Charlson comorbidity framework. The categories reported individually in the main manuscript were congestive heart failure; diabetes mellitus; diabetes with chronic complications; chronic pulmonary disease; renal disease; peripheral vascular disease; cerebrovascular disease; previous myocardial infarction; mild liver disease; moderate or severe liver disease; rheumatologic (connective tissue) disease; cancer history; metastatic solid tumor; dementia; peptic ulcer disease; hemiplegia or paraplegia; and AIDS/HIV. The ICD diagnosis codes used for each category are listed below.

Comorbidity category	ICD-10-CM	ICD-9-CM
Congestive heart failure	I099, I110, I130, I132, I255, I420, I425, I426, I427, I428, I429, I43, I50, P290	39891, 40201, 40211, 40291, 40401, 40403, 40411, 40413, 40491, 40493, 4254, 4255, 4256, 4257, 4258, 4259, 428
Diabetes mellitus	E100, E101, E106, E108, E109, E110, E111, E116, E118, E119, E120, E121, E126, E128, E129, E130, E131, E136, E138, E139, E140, E141, E146, E148, E149	2500, 2501, 2502, 2503, 2508, 2509
Diabetes with chronic complications	E102, E103, E104, E105, E107, E112, E113, E114, E115, E117, E122, E123, E124, E125, E127, E132, E133, E134, E135, E137, E142, E143, E144, E145, E147	2504, 2505, 2506, 2507
Chronic pulmonary disease	I278, I279, J40–J47, J60–J67, J684, J701, J703	4168, 4169, 490–505, 5064, 5081, 5088
Renal disease	I120, I131, N032–N037, N052–N057, N18, N19, N250, Z490–Z492, Z940, Z992	40301, 40311, 40391, 40402, 40403, 40412, 40413, 40492, 40493, 582, 5830–5837, 585, 586, 5880, V420, V451, V56
Peripheral vascular disease	I70, I71, I731, I738, I739, I771, I790, I792, K551, K558, K559, Z958, Z959	0930, 4373, 440, 441, 4431–4439, 4471, 5571, 5579, V434
Cerebrovascular disease	G45, G46, H340, I60–I69	36234, 430–438
Previous myocardial infarction	I21, I22, I252	410, 412
Mild liver disease	B18, K700–K703, K709, K713, K714, K715, K717, K73, K74, K760, K762, K763, K764, K768, K769, Z944	07022, 07023, 07032, 07033, 07044, 07054, 0706, 0709, 570, 571, 5733, 5734, 5738, 5739, V427
Moderate or severe liver disease	I850, I859, I864, I982, K704, K711, K721, K729, K765, K766, K767	4560, 4561, 4562, 5722–5728
Rheumatologic (connective tissue) disease	M05, M06, M315, M32, M33, M34, M351, M353, M360	4465, 7100–7104, 7140–7142, 7148, 725
Cancer history	C00–C26, C30–C34, C37–C41, C43, C45–C58, C60–C76, C81–C85, C88, C90–C97	140–172, 174–195, 200–208, 2386
Metastatic solid tumor	C77–C80	196–199
Dementia	F00–F03, F051, G30, G311	290, 2941, 3312
Peptic ulcer disease	K25–K28	531–534
Hemiplegia or paraplegia	G041, G114, G801, G802, G81, G82, G830–G834, G839	3341, 342, 343, 3440–3446, 3449
AIDS/HIV	B20, B21, B22, B24	042, 043, 044

D. Postoperative complications

The MAPBM postoperative-complication framework includes the following prespecified categories: acute kidney injury; acute respiratory distress syndrome; arrhythmia; cardiac arrest; cardiogenic pulmonary edema; deep-vein thrombosis; delirium; gastrointestinal bleeding; infection with uncertain source; laboratory-confirmed bloodstream infection; myocardial infarction; myocardial injury after non-cardiac surgery (MINS); pneumonia; paralytic ileus; postoperative hemorrhage; pulmonary embolism; stroke; surgical-site infection (superficial, deep, or organ/space); transfusion-related adverse events; urinary tract infection. For the present manuscript, the composite outcome was defined from diagnoses recorded during the index admission or during any readmission to the same hospital within 30 days after discharge. The ICD diagnosis codes used for each category are listed below; an asterisk indicates that all subcodes were included.

Complication category	ICD-10-CM	ICD-9-CM
Infection		
Surgical-site infection (superficial, deep, or organ/space)	T8130XA, T8140XA, T8141XA, T8142XA, T8143XA, T8149XA, T8450XA, T8451XA, T8452XA, T8453XA, T8454XA, T8459XA, T8460XA, T84620A, T84621A, T8469XA, T847XXA	99859, 99666
Urinary tract infection	N10, N390	5901*, 5990
Infection, source uncertain	R50* (excluding R5084)	7806* (excluding 78066)

Complication category	ICD-10-CM	ICD-9-CM
Laboratory-confirmed bloodstream infection	A41*, A207, A217, A227, A241, A267, A282, A287, A327, A377, A392, A393, A394, A427, A483, A5486, B007, R652*, R7881, T8144XA, T80211A	038*, 0031, 0049, 0200, 0223, 09889, 7907, 78552, 99590, 99591, 99592, 99932
Pneumonia	J12*, J13*, J14*, J15*, J16*, J17*, J18*	480*, 481*, 482*, 483*, 484*, 485*, 486*
Major adverse cardiac events (MACE)		
Arrhythmia	I44*, I47*, I48*, I49* (excluding I447, I481*, I482*)	426*, 427* (excluding 4263)
Cardiac arrest	I46*, I9712*, I9771*	4275, 9971
Cardiogenic pulmonary edema	I50*, I9713*, I97191, I97791, T8111XA (excluding I5022, I5032, I50812)	428*, 99801 (excluding 42822, 42832, 42842)
Myocardial infarction	I21*, I22*	41001, 41011, 41021, 41031, 41041, 41051, 41061, 41071, 41081, 41091
Myocardial injury after non-cardiac surgery (MINS)	I20*, I24*, I2511*	411*, 413*
Major adverse cardiac and cerebrovascular events (MACCE): MACE plus stroke		
Stroke	I63*, G45*, I6781, I6783, I97811, I97821	43311, 43321, 43331, 43381, 43391, 43401, 43411, 43491, 435*, 436, 4371
Other complications		
Acute kidney injury	N17*, N19*, N990	584*, 586
Acute respiratory distress syndrome	J80	51882
Delirium	F05	293*
Deep-vein thrombosis	I824*, I8281*, I82890, I8290, T8481XA, T8486XA	4534*, 4538*, 4539
Pulmonary embolism	I26*, T8481XA	415*
Gastrointestinal bleeding	K2289, K9162, K91841, K920, K921, K922	53082, 578*
Paralytic ileus	K560, K567	5601
Postoperative hemorrhage	G9732, G9752, I9742, I9762*, J9562, J95831, M96810, M96830, M96840, N9962, N99821, N99841, N99843, R58, T8483XA	4590, 99677, 99811, 99812
Transfusion-related adverse events	E8771, J9584, J9587, R5084, T800XXA, T801XXA, T80218A, T80219A, T8022XA, T8029XA, T8030XA, T80310A, T80311A, T80319A, T8039XA, T8040XA, T80410A, T80411A, T80419A, T8049XA, T8051XA, T8052XA, T8059XA, T8061XA, T8062XA, T8069XA, T80810A, T8089XA, T8090XA, T80910A, T80911A, T80919A, T8092XA, T80A0XA, T80A10A, T80A11A, T80A19A, T80A9XA	27661, 5187, 78066, 9996, 9997, 9998

Abbreviations: CABG, coronary artery bypass grafting; CVS, cardiac valve surgery; ESA, erythropoiesis-stimulating agent; Hb, hemoglobin; ICD, International Classification of Diseases; MAPBM, Maturity Assessment Model for Patient Blood Management; RBC, red blood cell; TAVI, transcatheter aortic valve implantation; TAVR, transcatheter aortic valve replacement.

Table S2. Sensitivity analysis of RBC transfusion rates according to the earliest available preoperative hemoglobin measurement within 120 days before surgery, documented preoperative treatment, and sex.

Hemoglobin stratum	Documented treatment	Women, n	Women, RBC transfusion, n (%)	Men, n	Men, RBC transfusion, n (%)	p-value
<12.0 g/dL	No documented intravenous iron or ESA	1,561	1,240 (79.4)	991	713 (71.9)	<0.001
	Intravenous iron only	227	188 (82.8)	192	140 (72.9)	0.014
	ESA only	21	20 (95.2)	34	29 (85.3)	0.390
	Intravenous iron + ESA	36	21 (58.3)	16	13 (81.2)	0.109
	**Subtotal**	**1,845**	**1,469 (79.6)**	**1,233**	**895 (72.6)**	**<0.001**
12.0–12.9 g/dL	No documented intravenous iron or ESA	2,056	1,433 (69.7)	1,002	564 (56.3)	<0.001
	Intravenous iron only	187	117 (62.6)	127	70 (55.1)	0.187
	ESA only	5	4 (80.0)	4	4 (100.0)	1.000
	Intravenous iron + ESA	9	6 (66.7)	10	6 (60.0)	1.000
	**Subtotal**	**2,257**	**1,560 (69.1)**	**1,143**	**644 (56.3)**	**<0.001**
≥13.0 g/dL	No documented intravenous iron or ESA	4,897	2,693 (55.0)	8,122	2,663 (32.8)	<0.001
	Intravenous iron only	237	134 (56.5)	262	80 (30.5)	<0.001
	ESA only	6	4 (66.7)	4	1 (25.0)	0.524

Hemoglobin stratum	Documented treatment	Women, n	Women, RBC transfusion, n (%)	Men, n	Men, RBC transfusion, n (%)	p-value
	Intravenous iron + ESA	5	3 (60.0)	6	2 (33.3)	0.567
	**Subtotal**	**5,145**	**2,834 (55.1)**	**8,394**	**2,746 (32.7)**	**<0.001**

Preoperative Hb for this sensitivity analysis was defined as the earliest available measurement within 120 days before surgery (N = 20,017; 9,247 women and 10,770 men). The earliest available Hb measurement was not necessarily obtained before administration of intravenous iron or an ESA. Data are presented as n (%). p-values compare RBC transfusion rates between women and men within each Hb stratum and treatment subgroup and were calculated using the chi-square test or Fisher's exact test, as appropriate. Treatment indication, dose, and hematologic response were unavailable, and the temporal relationship between treatment administration and the selected Hb measurement was not evaluated. Several subgroups contained few patients; treatment-stratified analyses should therefore be interpreted as exploratory. Abbreviations: ESA, erythropoiesis-stimulating agent; Hb, hemoglobin; RBC, red blood cell.

Table S3. Sensitivity analysis: adjusted sex differences in RBC transfusion within preoperative hemoglobin strata, using the earliest available hemoglobin measurement within 120 days before surgery.

Preoperative Hb stratum	Women, n	Men, n	Adjusted probability difference, percentage points (95% CI)	aOR (95% CI)	p-value
<12.0 g/dL	1,845	1,233	7.5 (3.8–11.1)	1.49 (1.24–1.78)	<0.001
12.0–12.9 g/dL	2,257	1,143	14.0 (10.1–17.9)	1.81 (1.54–2.12)	<0.001
≥13.0 g/dL	5,145	8,394	21.5 (19.1–23.9)	2.53 (2.33–2.73)	<0.001

Estimates compare women with men within the same Hb stratum and were derived from the same mixed-effects logistic regression model as Table 3 of the main manuscript (sex, Hb category, and their interaction), adjusted for age, comorbidity burden, surgical complexity, valve position, and year of surgery, with a random intercept for center. Hb strata were defined using the earliest available measurement within 120 days before surgery (N = 20,017; 9,247 women and 10,770 men). Overall sex × Hb category interaction: $p < 0.001$. Abbreviations: aOR, adjusted odds ratio; CI, confidence interval; Hb, hemoglobin; RBC, red blood cell.

Table S4. Patients with and without an available preoperative hemoglobin measurement, before and after propensity-score matching.

	Before matching			After matching		
Characteristic	No Hb (n = 7,811)	Hb available (n = 20,017)	SMD / p-value	No Hb (n = 2,070)	Hb available (n = 2,070)	SMD / p-value
Age, years, mean (SD)	65.5 (15.6)	65.8 (14.1)	−0.02	68.5 (13.0)	68.5 (13.0)	0.00
Women, n (%)	3,600 (46.1)	9,247 (46.2)	0.00	998 (48.2)	967 (46.7)	0.03
Comorbidity burden, n (%)						
0	3,526 (45.1)	8,738 (43.7)	0.03	857 (41.4)	852 (41.2)	0.00
1–2	3,764 (48.2)	9,897 (49.4)	−0.03	1,085 (52.4)	1,062 (51.3)	0.02
3–4	505 (6.5)	1,313 (6.6)	0.00	126 (6.1)	155 (7.5)	−0.06
≥5	16 (0.2)	69 (0.3)	−0.03	2 (0.1)	1 (0.0)	0.02
Surgical complexity, n (%)						
Single-valve procedure	6,912 (88.5)	17,545 (87.7)	0.03	1,916 (92.6)	1,912 (92.4)	0.01
Double-valve procedure	848 (10.9)	2,360 (11.8)	−0.03	146 (7.1)	153 (7.4)	−0.01
Triple-valve procedure	51 (0.7)	112 (0.6)	0.01	8 (0.4)	5 (0.2)	0.03
Outcomes (p-value)						
RBC transfusion, n (%)	2,425 (31.0)	10,148 (50.7)	<0.001	692 (33.4)	1,117 (54.0)	<0.001
Any postoperative complication, n (%)	2,936 (37.6)	8,002 (40.0)	<0.001	838 (40.5)	888 (42.9)	0.122
Hospital length of stay, days, mean (SD)	13.5 (13.0)	13.6 (12.3)	0.328	13.8 (13.0)	15.1 (14.0)	<0.001
In-hospital mortality within 30 days, n (%)	265 (3.4)	591 (3.0)	0.069	70 (3.4)	71 (3.4)	0.998

The comparison is restricted to the 32 centers that reported laboratory data; a further 8 centers (1,929 eligible patients) reported no preoperative hemoglobin at all and were therefore excluded from this analysis. Patients were matched 1:1 within center on the propensity score for having no available hemoglobin measurement (age, sex, comorbidity burden, surgical complexity and year of surgery), yielding 2,070 matched pairs. The last column of each block reports the p-value (chi-square test or Mann–Whitney U test) for outcomes and standardized mean difference for baseline characteristics. After matching, postoperative complication rates and in-hospital mortality within 30 days did not differ significantly between groups; however, RBC transfusion remained more frequent among patients with available preoperative Hb (54.0% vs. 33.4%; p <

0.001), and hospital length of stay remained longer (15.1 vs. 13.8 days; $p < 0.001$). Abbreviations: Hb, hemoglobin; RBC, red blood cell; SD, standard deviation; SMD, standardized mean difference.

Supplementary Figure S1. Between-center variability in red blood cell transfusion and postoperative complications.

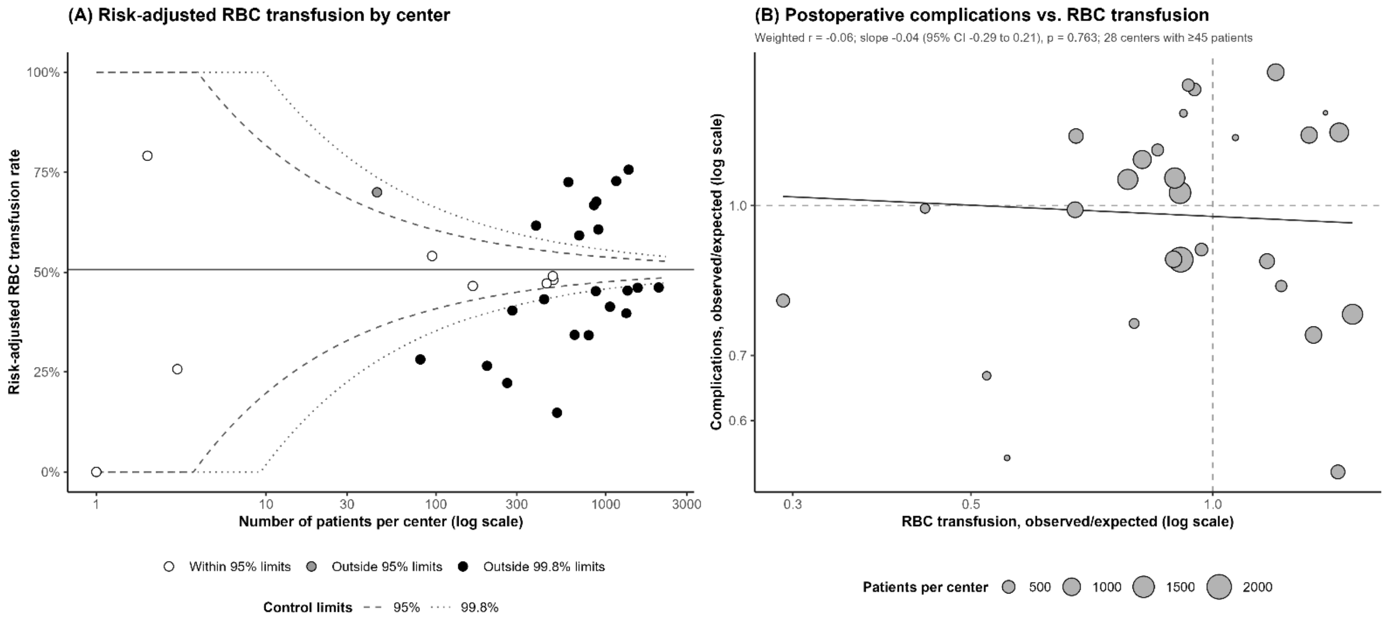

(A) Funnel plot showing risk-adjusted RBC transfusion rates across participating centers, with 95% and 99.8% control limits. (B) Association between center-level observed-to-expected ratios for RBC transfusion and postoperative complications. Bubble size represents the number of patients per center. The analysis was restricted to 28 centers with at least 45 patients. No statistically significant association was observed (patient-volume-weighted slope, −0.04; 95% CI, −0.29 to 0.21; p = 0.763).

Checklist S5. STROBE Statement—checklist of items that should be included in reports of observational studies

	**Item No.**	**Recommendation**	**Page No.**	**Relevant text from manuscript**
**Title and abstract**	1	(*a*) Indicate the study's design with a commonly used term in the title or the abstract	Abstract	Design stated in the abstract: nationwide multicenter retrospective cohort study.
		(*b*) Provide in the abstract an informative and balanced summary of what was done and what was found	Abstract	Structured abstract (Background/Objectives, Methods, Results, Conclusions).
**Introduction**				
Background/rationale	2	Explain the scientific background and rationale for the investigation being reported	Introduction	Sex differences in transfusion after cardiac surgery; WHO vs PBM-oriented anemia definitions; gaps in current evidence.

Objectives	3	State specific objectives, including any prespecified hypotheses	Introduction, last paragraph	Primary and secondary aims, exploratory treatment analysis, and prespecified hypothesis.
**Methods**				
Study design	4	Present key elements of study design early in the paper	Methods: Study design and data source	Nationwide multicenter retrospective cohort using the MAPBM registry; reported following STROBE and SAGER.
Setting	5	Describe the setting, locations, and relevant dates, including periods of recruitment, exposure, follow-up, and data collection	Methods: Study design and data source; Outcomes	40 Spanish hospitals, January 2016 to December 2024. Outcome ascertainment included index-admission records and readmissions to the same hospital, with outcome-specific follow-up windows. In-hospital mortality was restricted to the first 30 days after initial admission.
Participants	6	(*a*) *Cohort study*—Give the eligibility criteria, and the sources and methods of selection of participants. Describe methods of follow-up<br><br>*Case-control study*—Give the eligibility criteria, and the sources and methods of case ascertainment and control selection. Give the rationale for the choice of cases and controls<br><br>*Cross-sectional study*—Give the eligibility criteria, and the sources and methods of selection of participants	Methods: Study population; Table S1	Adults undergoing elective open cardiac valve surgery identified by ICD codes. Exclusions: non-elective surgery, concomitant CABG or heart transplantation, endocarditis, unknown surgical complexity. Follow-up from registry hospital records.
		(*b*) *Cohort study*—For matched studies, give matching criteria and number of exposed and unexposed<br><br>*Case-control study*—For matched studies, give matching criteria and the number of controls per case	Not applicable	Not a matched cohort. Propensity-score matching was used only to compare patients with and without an available Hb measurement (Table S4).
Variables	7	Clearly define all outcomes, exposures, predictors, potential confounders, and effect modifiers. Give diagnostic criteria, if applicable	Methods: Study population; Preoperative hemoglobin assessment; Preoperative anemia treatment; Outcomes	Exposure: preoperative Hb. Effect modifier: sex. Confounders: age, comorbidity burden, surgical complexity, valve position, year of surgery, center. Outcomes: RBC transfusion, postoperative complications, length of

				stay, in-hospital mortality within 30 days.
Data sources/ measurement	8*	For each variable of interest, give sources of data and details of methods of assessment (measurement). Describe comparability of assessment methods if there is more than one group	Methods: Study design and data source; Study population; Table S1	Registry data integrating laboratory, pharmacy, transfusion, surgical and administrative records; ICD-based definitions of eligibility, comorbidities and complications.
Bias	9	Describe any efforts to address potential sources of bias	Methods: Statistical analysis; Results; Tables S2–S4; Discussion	Multivariable mixed-effects models with random center intercept; sensitivity analysis using the earliest Hb; comparison of patients with and without Hb before and after propensity-score matching; limitations discussed.
Study size	10	Explain how the study size was arrived at	Results; Figure 1	All eligible patients in the registry during the study period were included; no a priori sample size calculation.
Quantitative variables	11	Explain how quantitative variables were handled in the analyses. If applicable, describe which groupings were chosen and why	Methods: Preoperative hemoglobin assessment; Study population	Hb analyzed continuously and in three prespecified categories (<12.0, 12.0–12.9, ≥13.0 g/dL); comorbidity burden grouped as 0, 1–2, 3–4 or ≥5 Charlson categories.
Statistical methods	12	(*a*) Describe all statistical methods, including those used to control for confounding	Methods: Statistical analysis	Mixed-effects logistic and Poisson regression adjusted for age, comorbidity burden, surgical complexity, valve position, and year of surgery, with a random intercept for center.
		(*b*) Describe any methods used to examine subgroups and interactions	Methods: Statistical analysis	Sex × Hb interaction in categorical (likelihood-ratio test) and continuous models; exploratory treatment-stratified analysis.
		(*c*) Explain how missing data were addressed	Methods: Preoperative hemoglobin assessment; Statistical	Complete-case analysis without imputation; patients without an available Hb

			analysis; Table S4	measurement compared with the analyzed cohort.
		(*d*) *Cohort study*—If applicable, explain how loss to follow-up was addressed<br><br>*Case-control study*—If applicable, explain how matching of cases and controls was addressed<br><br>*Cross-sectional study*—If applicable, describe analytical methods taking account of sampling strategy	Methods: Outcomes; Discussion	Follow-up based on hospital records; deaths after discharge outside the hospital were not captured (acknowledged as a limitation).
		(*e*) Describe any sensitivity analyses	Methods: Preoperative hemoglobin assessment; Tables S2–S4	Earliest available Hb within 120 days before surgery; propensity-score matched comparison of patients with and without Hb.
**Results**				
Participants	13*	(a) Report numbers of individuals at each stage of study—eg numbers potentially eligible, examined for eligibility, confirmed eligible, included in the study, completing follow-up, and analysed	Results; Figure 1	30,564 patients identified, 29,757 eligible and 20,017 included in Hb-based analyses.
		(b) Give reasons for non-participation at each stage	Results; Figure 1	Number excluded at each step with reasons; 9,740 eligible patients without an available preoperative Hb.
		(c) Consider use of a flow diagram	Figure 1	Flow chart of patient selection and exclusion.
Descriptive data	14*	(a) Give characteristics of study participants (eg demographic, clinical, social) and information on exposures and potential confounders	Results; Table 1	Demographic and clinical characteristics, stratified by sex.
		(b) Indicate number of participants with missing data for each variable of interest	Results; Table S4	Preoperative Hb unavailable in 9,740 of 29,757 eligible patients (32.7%); records with unknown surgical complexity excluded.
		(c) *Cohort study*—Summarise follow-up time (eg, average and total amount)	Methods: Outcomes; Table 1	Follow-up was based on the index admission and recorded readmissions to the same hospital. In-hospital mortality was assessed within 30 days of the initial admission.
Outcome data	15*	*Cohort study*—Report numbers of outcome events or summary measures over time	Results; Tables 1–2	Numbers of RBC transfusions, postoperative complications, and in-hospital deaths within 30

				days, as well as hospital length of stay, by sex; transfusion according to Hb stratum and documented treatment.
		Case-control study—Report numbers in each exposure category, or summary measures of exposure	Not applicable	Cohort study.
		Cross-sectional study—Report numbers of outcome events or summary measures	Not applicable	Cohort study.
Main results	16	(*a*) Give unadjusted estimates and, if applicable, confounder-adjusted estimates and their precision (eg, 95% confidence interval). Make clear which confounders were adjusted for and why they were included	Results; Tables 1–4; Figures 2–3	Unadjusted rates; adjusted probability differences and aORs with 95% CIs. Adjustment variables listed in Statistical analysis.
		(*b*) Report category boundaries when continuous variables were categorized	Methods: Preoperative hemoglobin assessment	Hb categories: <12.0, 12.0–12.9 and ≥13.0 g/dL.
		(*c*) If relevant, consider translating estimates of relative risk into absolute risk for a meaningful time period	Results; Table 3; Figure 2	Adjusted absolute differences in transfusion probability (percentage points).
Other analyses	17	Report other analyses done—eg analyses of subgroups and interactions, and sensitivity analyses	Results; Table 2; Tables S2–S4; Supplementary Figure S1	Sex × Hb interactions, treatment-stratified analyses and sensitivity analyses, and exploratory analysis of between-center variability in transfusion and postoperative complications.
**Discussion**				
Key results	18	Summarise key results with reference to study objectives	Discussion, first paragraph	Summary of the main findings in relation to the study aims.
Limitations	19	Discuss limitations of the study, taking into account sources of potential bias or imprecision. Discuss both direction and magnitude of any potential bias	Discussion, last paragraph	Residual confounding, missing Hb, unmeasured perioperative variables, unmeasured procedural characteristics and residual surgical confounding, administrative coding, mortality ascertainment and treatment data.
Interpretation	20	Give a cautious overall interpretation of results considering objectives, limitations, multiplicity of	Discussion; Conclusions	Findings interpreted as supporting risk recognition rather than a

		analyses, results from similar studies, and other relevant evidence		therapeutic target; comparison with previous studies.
Generalisability	21	Discuss the generalisability (external validity) of the study results	Discussion, last paragraph	Cohort limited to Spanish hospitals in a national PBM benchmarking programme; generalizability may be limited.
**Other information**				
Funding	22	Give the source of funding and the role of the funders for the present study and, if applicable, for the original study on which the present article is based	Funding	This research received no external funding.

*Give information separately for cases and controls in case-control studies and, if applicable, for exposed and unexposed groups in cohort and cross-sectional studies.

Note: An Explanation and Elaboration article discusses each checklist item and gives methodological background and published examples of transparent reporting. The STROBE checklist is best used in conjunction with this article (freely available on the Web sites of PLoS Medicine at http://www.plosmedicine.org/, Annals of Internal Medicine at http://www.annals.org/, and Epidemiology at http://www.epidem.com/). Information on the STROBE Initiative is available at www.strobe-statement.org.
